# Interrelated modulation of endothelial function in Behcet's disease by clinical activity and corticosteroid treatment

**DOI:** 10.1186/ar2289

**Published:** 2007-09-11

**Authors:** Athanase D Protogerou, Petros P Sfikakis, Kimon S Stamatelopoulos, Christos Papamichael, Kostas Aznaouridis, Emmanuil Karatzis, Theodore G Papaioannou, Ignatios Ikonomidis, Phedon Kaklamanis, Myron Mavrikakis, John Lekakis

**Affiliations:** 1 Vascular Laboratory, Department of Clinical Therapeutics, Alexandra Hospital, Medical School, University of Athens, V. Sofias,115 28, Athens, Greece; 2 1^st ^Department of Propeudeutic and Internal Medicine, Laikon Hospital, Medical School, University of Athens, Ag. Thoma, 115 27, Athens, Greece; 3 2^nd ^Department of Cardiology, Attikon Hospital, Medical School, University of Athens, Rimini, 124 61, Athens, Greece

## Abstract

Corticosteroids are commonly used in empirical treatment of Behçet's disease (BD), a systemic inflammatory condition associated with reversible endothelial dysfunction. In the present study we aimed to dissect the effects of clinical disease activity and chronic or short-term corticosteroid treatment on endothelial function in patients with BD. In a case-control, cross-sectional study, we assessed endothelial function by endothelium dependent flow mediated dilatation (FMD) at the brachial artery of 87 patients, who either were or were not receiving chronic corticosteroid treatment, and exhibiting variable clinical disease activity. Healthy individuals matched for age and sex served as controls. Endothelial function was also assessed in a prospective study of 11 patients before and after 7 days of treatment with prednisolone given at disease relapse (20 mg/day). In the cross-sectional component of the study, FMD was lower in patients than in control individuals (mean ± standard error: 4.1 ± 0.4% versus 5.7 ± 0.2%, *P *= 0.003), whereas there was a significant interaction between the effects of corticosteroids and disease activity on endothelial function (*P *= 0.014, two-factor analysis of variance). Among patients with inactive BD, those who were not treated with corticosteroids (*n *= 33) had FMD comparable to that in healthy control individuals, whereas those treated with corticosteroids (*n *= 15) had impaired endothelial function (*P *= 0.023 versus the respective control subgroup). In contrast, among patients with active BD, those who were not treated with corticosteroids (*n *= 20) had lower FMD than control individuals (*P *= 0.007), but in those who were receiving corticosteroids (*n *= 19) the FMD values were comparable to those in control individuals. Moreover, FMD was significantly improved after 7 days of prednisolone administration (3.7 ± 0.9% versus 7.6 ± 1.4%, *P *= 0.027). Taken together, these results imply that although corticosteroid treatment may impair endothelial function *per se *during the remission phase of the inflammatory process, it restores endothelial dysfunction during active BD by counteracting the harmful effects of relapsing inflammation.

## Introduction

Behçet's disease (BD) is a relapsing systemic inflammatory condition of unknown aetiology that is more prevalent in certain geographical areas and particular ethnic groups [1,2]. Multiple immunological abnormalities, which are possibly induced by microbial antigens in genetically susceptible individuals, appear to be important in the pathogenesisof BD. Such abnormalities are related to the enhanced inflammatory response observed in these patients, and endothelial activation and injury and the resultant occlusive vasculopathy may also contribute to the tissue damage [2]. Large vessel involvement is common [3], and vascular complications are among the leading causes of increased morbidity and mortality [4]. Although the optimal treatment of these complications is subject to debate, the current empirical approach includes high doses of corticosteroids [5]. Early identification of those patients who are at high risk for developing severe vasculopathy and in need of aggressive treatment remains difficult, and development of means to achieve early identification is a great challenge in this area.

Multiple lines of evidence indicate that surrogate vascular markers, such as endothelium dependent flow mediated dilatation (FMD), arterial stiffness and arterial wall thickness, are strong predictors of cardiovascular risk in various cardiovascular conditions. We and others have previously shown that endothelial function, assessed noninvasively by high resolution ultrasound, is impaired in patients with BD [6-9]. Although inflammation is a well described modulator of endothelial function [10] and is the target of corticosteroid treatment, which is commonly used in the management of various manifestations of BD [1,3,5], little is known about the effects of corticosteroid treatment on endothelial function in patients with chronic inflammatory diseases. Interestingly, administration of corticosteroids improves endothelial function in patients with giant cell arteritis, which is the most common primary vasculitic disease [11]. Conversely, corticosteroids may exert detrimental effects on endothelial function in the absence of an inflammatory condition [12,13].

The present study was conducted, using both case-controlled cross-sectional and prospective interventional approaches, to dissect the effects of clinical disease activity and chronic or short-term corticosteroid treatment on endothelial function in patients with BD.

## Materials and methods

### Cross-sectional, case-control study

Consecutive patients, who were regularly examined in our clinics and had an established diagnosis of BD (according to the International Study Group criteria [12]), were referred to the vascular laboratory within 3 to 5 days after they had their last clinical evaluation to determine clinical disease status. Exclusion criteria included diabetes mellitus, concomitant infection and treatment with anti-tumour necrosis factor agents. From a total of 87 referred patients (aged 17 to 71 years) 39 were classified as having active disease, defined by the presence of more than two clinical characteristics, including the following [14]: oral ulcers, genital ulcers, erythema nodosum, pseudophollicullitis and ocular lesions. On the day of the vascular studies, patients with active BD were receiving no treatment (*n *= 3), corticosteroids (mean dose equivalent to 20 mg/day; *n *= 19), azathioprine (*n *= 6), cyclosporine A (*n *= 4), or colchicines (*n *= 23), or a combination of these agents. Any suggested drug modifications at their last clinical evaluation had been immediately adopted (3 to 5 days before the vascular studies). The remaining patients were classified as having stable or inactive disease (*n *= 48), receiving steady medication for at least 1 month (corticosteroids equivalent to 2.5 to 7.5 mg/day, *n *= 15; azathioprine, *n *= 7; cyclosporine A, *n *= 8; colchicine, *n *= 24). The control group included 87 healthy individuals, matched for age and sex, who were studied in parallel. None of the patients or control individuals had a history of stroke or coronary heart disease.

### Prospective interventional study

Eleven of the 87 patients described above (five were male; age 33 to 54 years) who presented at disease relapse while not receiving corticosteroid treatment were studied prospectively. Subsequent to their clinical assessment, these patients underwent vascular studies (day 0) and they were prescribed a daily regimen of 20 mg prednisolone (according to standard clinical practice for disease relapse), in addition to concomitant treatment, which remained unchanged during the 7 following days. Clinical assessment and vascular studies were repeated on day 7.

### Brachial artery reactivity

Endothelial function was studied, noninvasively, by means of brachial artery FMD, as described previously [15]. Patients and control individuals abstained from consumption of tobacco, coffee, alcohol, antioxidant-containing beverages and medications for 8 hours before the vascular studies, which were always performed during the morning. Premenopausal women were examined during any day of their menstrual cycle except from the M phase. A high-resolution ultrasound system (7.0 MHz; Accuson 128XP/10, Accuson, Mountain View, CA, USA) was used. In brief, after 10 min of rest in a supine position in a quiet, temperature controlled room (21°C to 23°C), the brachial artery was scanned longitudinally and its diameter at end-diastole (from the inner border line of adventitia to adventitia) was measured. Subsequently, 5 min of ischaemia was induced by an inflated cuff (250 mmHg), fitted at 8 cm distal to the brachial artery, near the wrist. During reactive hyperaemia (60 to 90 s after cuff deflation) the vessel's maximal diameter, at exactly the same anatomical site, was measured. FMD was then calculated as the percentage increase in diameter from baseline. After 10 min, a second scan at rest was performed and then nitroglycerin was administered (400 μg sublingual); 5 min later a final scan was taken in order to assess nitrate mediated dilatation (NMD), which was used as an index of endothelium independent vasodilatation, in order to test the functional status of the arterial wall smooth muscle.

All participants gave informed consent and the protocol was approved by the Alexandra Hospital's research ethics committee.

### Statistical analysis

Statistical analyses were performed using SPSS (13.0 version; SPSS Inc., Chicago, IL, USA). χ^2 ^tests were used to compare qualitative variables between groups. All of the quantitative variables had a normal distribution curve. In the cross-sectional study analysis of variance (ANOVA), provided by the general linear model, was applied to the comparison of mean values between subgroups. Analysis by *t*-test was applied for pair-wise comparisons, when appropriate. Two-factor ANOVA was also used in the cross-sectional study to investigate the presence of potential interaction between the presence of active disease (as defined above) and corticosteroid treatment (activity × corticosteroids) on FMD. In the prospective study, because of the limited number of patients, nonparametric analysis for two related samples (Wilkoxon test) was used to compare changes in FMD and NMD, before and after 7 days on corticosteroid treatment. Values are expressed as mean ± standard error of the mean. *P *< 0.05 was deemed to indicate statistical significance.

## Results

### Effects of disease activity and corticosteroid treatment on endothelial function

There were no significant differences in cardiovascular risk factors known to affect endothelial function between healthy control individuals (mean values shown in Table 1) and patients with BD (*n *= 87; data not shown for the whole group), except for body mass index (*P *< 0.05), which was higher in the control group. Endothelium dependent FMD was lower in patients than in control individuals (4.1 ± 0.4% versus 5.7 ± 0.4%, respectively; *P *= 0.003), whereas endothelium independent NMD was similar between groups (12.8 ± 0.5% versus 13.4 ± 0.7%; not significant). Adjustment for body mass index did not modify these results.

**Table 1:** Cardiovascular risk factors

	Control individuals (*n *= 87)	Corticosteroids (-)		Corticosteroids (+)	
		
		Active BD (*n *= 20)	Stable or inactive BD (*n *= 33)	Active BD (*n *= 19)	Stable or inactive BD (*n *= 15)
Males/females (*n*)	58/32	13/7	22/11	14/5	9/6
Age (years)	40.1 ± 1.2	40.9 ± 2.5	40.6 ± 1.5	38.4 ± 2.5	37.6 ± 2.9
Hypertension (*n *[%])	13 (14.9)	2 (10.1)	3 (9.4)	5 (26.3)	3 (20.0)
Smokers (*n *[%])	40 (46)	12 (60.0)	18 (54.4)	13 (68.4)	5 (33.3)
BMI (kg/m^2^)	27.2 ± 0.7	23.5 ± 1.3	25.2 ± 0.6	24.7 ± 1.0	23.4 ± 1.1
Total cholesterol (mg/dl)	193.2 ± 5.3	200.7 ± 16.0	202.7 ± 8.3	209.6 ± 14.1	215.4 ± 12.2
LDL-cholesterol (mg/dl)	132.4 ± 4.5	120.1 ± 15.1	132.2 ± 7.8	134.6 ± 4.9	143.5 ± 11.2
HDL-cholesterol (mg/dl)	47.7 ± 1.4	44.7 ± 5.5	46.8 ± 2.5	52.1 ± 4.5	52.4 ± 4.1
Triglycerides (mg/dl)	106.6 ± 9.9	115.5 ± 32.2	124.1 ± 12.5	133.2 ± 25.3	125.4 ± 22.8
Glucose (mg/dl)	85.3 ± 1.5	94.1 ± 4.4	91.3 ± 2.0	89.9 ± 3.7	84.5 ± 3.5

Shown are cardiovascular risk factors in control individuals and in patients with Behçet's disease (BD) divided according to the receipt (+) or nonreceipt (-) of corticosteroid treatment and the clinical disease status. Unless otherwise stated, values are expressed as mean ± standard error. BMI, body mass index; HDL, high-density lipoprotein; LDL, low-density lipoprotein.

FMD was similar between patients receiving corticosteroids (*n *= 34; 4.3 ± 0.6%) and patients not receiving corticosteroids (*n *= 53; 4.0 ± 0.5%). Patients with active BD (*n *= 39) had higher FMD than did patients with inactive BD (*n *= 48; 4.5 ± 0.6% versus 3.8 ± 0.6%, respectively), although this did not reach statistical significance. Two-factor ANOVA revealed a significant interaction between the effect of corticosteroids and disease activity on endothelial function (*P *= 0.014). These results were unchanged following adjustment for cardiovascular risk factors and after excluding those patients who were receiving treatment with cyclosporine A and azathioprine (data not shown).

**Figure 1:**
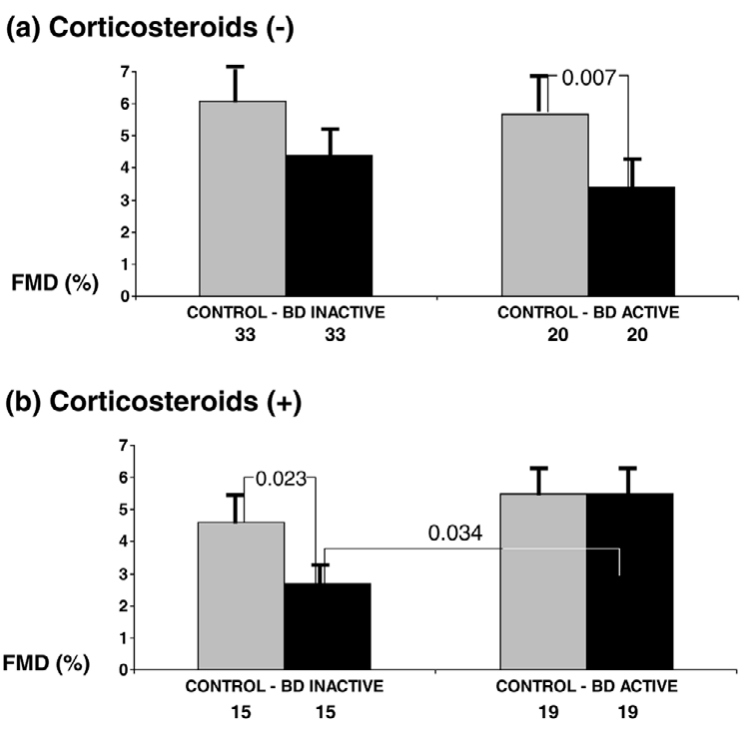
Endothelial function in patients with BD and control individuals (cross sectional study). Shown is endothelium-dependent flow mediated dilatation (FMD) at the brachial artery in subgroups of patients with active or inactive Behçet's disease (BD) subdivided according to **(a) **absence or **(b) **presence of corticosteroid treatment, and their respective control subgroups. Numbers of patients and significant differences between subgroups (*t*-test) are shown. Values are expressed as mean ± standard error.

Subsequently, FMD was analyzed among the four subgroups of patients defined by the presence of active or stable/inactive disease and the receipt or nonreceipt of corticosteroid treatment, as compared with the FMD in respective matched control subgroups (Figure 1). Although specific matching for cardiovascular risk factors (including history of hypertension and smoking) was not performed, no significant differences were found in those parameters between control subgroups (data not shown) and patient subgroups (Table 1). ANOVA revealed that among patients not receiving corticosteroid treatment, and in comparison with the control subgroups, FMD was low in those with stable or inactive disease and even lower in those with active disease (*P *= 0.006). Further analysis revealed a significant difference only between patients with active BD and the control subgroup (*P *= 0.007; Figure 1a). However, among corticosteroid-treated patients, FMD was comparable to that in control individual when only those with active disease were considered, but it was lower than that in control individuals when only those with stable/inactive disease were considered (*P *< 0.04, ANOVA). Further pair-wise comparisons revealed significant differences only between patients with stable/inactive BD and the control subgroup (*P *= 0.023; Figure 1b).

Furthermore, Figure 1 shows that among patients receiving corticosteroids, those with active disease had higher FMD than did those with inactive disease (5.5 ± 1.0% versus 2.7 ± 0.7%; *P *= 0.034). Those patients with active disease receiving corticosteroids tended to have higher FMD than did those not receiving corticosteroids (5.5 ± 1.0% versus 3.4 ± 0.6%; *P *= 0.109), whereas those patients with inactive disease on corticosteroids tended to have lower FMD than did those who were not receiving corticosteroids (2.7 ± 0.7% versus 4.4 ± 0.6%; *P *= 0.096).

Regarding NMD, no differences were found between active and inactive BD or between corticosteroid receiving and nonreceiving patients, and no interaction between corticosteroids and BD disease was identified (data not shown).

### Short-term prednisolone treatment upon relapse improves endothelial function

**Figure 2:**
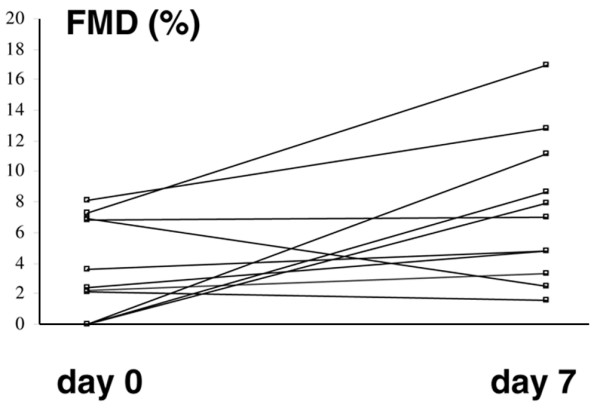
Endothelial function in patients with BD (prospective study). Shown are changes in endothelium dependent flow mediated dilatation (FMD) at the brachial artery, in 11 patients with Behçet's disease (BD), before and after 7 days of treatment with prednisolone (20 mg/day).

Eleven patients of those who were not treated with corticosteroids were studied upon disease relapse (signs of relapse: oral ulcers, *n *= 9; erythema nodosum, *n *= 7; venous thrombosis, *n *= 2; genital ulcers, *n *= 2; arthritis, *n *= 4, and central nervous system involvement, *n *= 1), as well as after 7 days of administration of prednisolone (20 mg/day). As shown in Figure 2 endothelial function improved from days 0 to 7 in eight patients and remained unchanged or decreased in the three remaining patients. Endothelium dependent FMD increased significantly from days 0 to 7 (3.7 ± 0.9% versus 7.6 ± 1.4%; *P *= 0.027), whereas endothelium independent dilatation, as assessed by considering NMD, was not significantly affected (15.2 ± 1.2% versus 16.4 ± 1.1%; not significant). All patients had a partial remission of their symptoms at day 7.

## Discussion

The main finding of the cross-sectional observational study was that endothelial function in patients with BD is modulated by both clinical disease activity and use of corticosteroids, albeit in an interrelated manner. Because the degree of inflammation cannot safely be assessed in BD using surrogate markers, such as serum C-reactive protein levels (which do not correlate with the clinical activity [1]), disease status was assessed by clinical means only. Endothelial dysfunction was assessed by a widely-used, reproducible and non-invasive method, i.e. the FMD at a medium-size conduit artery. FMD is considered by definition as a reversible disturbance and it is an independent predictor of cardiovascular events [15]. FMD has been proved to be mainly induced by production of nitric oxide from endothelial cells [15], and higher values correlate with better functional status of the endothelium. It was previously shown that low-grade, acute inflammatory response, such as the response induced by *Salmonella typhi *vaccine, may lead to a significant detrimental effect on endothelial function (FMD), albeit one that is reversible [10]. More recent data demonstrated that short-term systemic inflammation, induced by intensive periodontal treatment, resulted in acute endothelial dysfunction (FMD), although this was reversible by 6 months after therapy [16]. Finally, in a small group of patients with active BD, endothelial function was improved by short-term administration of antioxidants [6]. This further implies that there exists a connection between inflammation, oxidative stress and endothelial function [17,18], at least in the acute inflammatory phase, which may be associated with the ability of glucocorticoids to modulate nitric oxide synthase production [18].

By studying a large group of patients and control individuals with comparable cardiovascular risk, a significant effect of BD disease activity and corticosteroid treatment on endothelial function was indeed identified. It was also shown that these results were not modified by the presence of other immunosuppressive drugs. Taken together with the presence of the significant interaction between BD activity and corticosteroid treatment, the results obtained in the cross-sectional part of the study suggest that in the presence of higher levels of systemic inflammation endothelial dysfunction may be prevented by corticosteroid treatment. Moreover, the results of the prospective interventional study showed that the endothelial function was improved after treatment with 20 mg/day prednisolone given at disease relapse. A similarly beneficial corticosteroid-induced effect during the inflammatory phase has also been observed in patients with newly diagnosed giant cell arteritis [11]. Endothelium independent mechanisms did not account for our findings, because NMD was unaffected by clinical activity of BD or corticosteroid treatment in either the cross-sectional or the prospective study. These results might partly explain the beneficial effects of corticosteroids on vascular complications in patients with BD [3,5]. Whether the level of endothelial dysfunction (as indicated by FMD) in individual patients may predict future adverse vascular events in BD requires further investigation.

On the other hand, the results of the case-control cross-sectional study imply that the chronic use of corticosteroids may impair endothelial function in patients with stable or inactive disease and thus lower inflammatory burden. As previously described in healthy individuals, corticosteroid use may induce an unfavourable effect on endothelial function in the absence of systemic inflammation [13]. Increased oxidative stress and endothelial dysfunction have been described in patients with endogenous hypercortisolaemia in the absence of a concomitant inflammatory process [12]. This effect may be attributed to decreased stability of the nitric oxide synthase, increased oxidative stress, or increased endothelin-1 production [12,19,20]. The presence of and the role played by endothelial dysfunction in the atherosclerotic process, as well as its reversibility, have been confirmed in various clinical settings, including systemic vasculitis, rheumatoid arthritis, systemic lupus erythematosous and systemic sclerosis [21-25]. However, the optimal dose and duration of corticosteroid treatment with respect to endothelial function related effects are not known.

## Conclusion

The findings presented here suggest that corticosteroids have a bipolar effect on endothelial function in BD depending on the level of inflammatory burden. Whether endothelial function during the remission phase of the inflammatory process may deteriorate with chronic corticosteroid use has not previously been addressed and should be confirmed in a prospective manner. Such studies should also address the question of potential dose-effect differences of corticosteroid treatment with respect to endothelial function, because the mean dose of corticosteroids used in the inactive patients in the cross-sectional study was considerably less than that used among the patients with active BD, both in the cross-sectional and in the prospective parts of our study. Nevertheless, the study shows that corticosteroid treatment, which is commonly prescribed in acute relapses of BD and especially for complications that involve the large vessels [3-5], may restore endothelial dysfunction by counteracting the harmful effects of relapsing inflammation.

## Abbreviations

ANOVA = analysis of variance; BD = Behçet's disease; FMD = flow mediated dilatation; NMD = nitrate mediated dilatation.

## Competing interests

The authors declare that they have no competing interests.

## Authors' contributions

ADP conceived of the study, participated in its design and wrote the manuscript. PPS participated in designing the study and helped to revise the manuscript. KSS performed haemodynamic measurements. CP participated in sequence alignment and drafted the manuscript. KA performed haemodynamic measurements. EK drafted the manuscript. TGP performed the statistical analysis. II drafted the manuscript. PK participated in designing the study and helped to revise the manuscript. MM participated in designing the study and helped to revise the manuscript. JL participated in designing the study and helped to revise the manuscript. All authors read and approved the final manuscript, and made substantial contributions to it.
